# Switch to Fixed Dose of Doravirine, Lamivudine, Tenofovir Disoproxil Fumarate Versus Bictegravir, Emtricitabine, and Tenofovir Alafenamide Fumarate in Virologically Suppressed Adults on Efavirenz-Based Regimens: 48-Week Results of a Real-world, Prospective, Observational Cohort Study

**DOI:** 10.1093/ofid/ofaf808

**Published:** 2026-01-20

**Authors:** Aixin Li, Zaicun Li, Jianwei Li, Yue Gao, Lili Dai, An Liu, Hongwei Zhang, Xi Wang, Liang Wu, Yanwei Yao, LetiAn Liu, Jiangzhu Ye, Lijun Sun

**Affiliations:** Center for Infectious Diseases, Beijing Youan Hospital, Capital Medical University, Beijing, People's Republic of China; Center for Infectious Diseases, Beijing Youan Hospital, Capital Medical University, Beijing, People's Republic of China; Center for Infectious Diseases, Beijing Youan Hospital, Capital Medical University, Beijing, People's Republic of China; Center for Infectious Diseases, Beijing Youan Hospital, Capital Medical University, Beijing, People's Republic of China; Center for Infectious Diseases, Beijing Youan Hospital, Capital Medical University, Beijing, People's Republic of China; Center for Infectious Diseases, Beijing Youan Hospital, Capital Medical University, Beijing, People's Republic of China; Center for Infectious Diseases, Beijing Youan Hospital, Capital Medical University, Beijing, People's Republic of China; Center for Infectious Diseases, Beijing Youan Hospital, Capital Medical University, Beijing, People's Republic of China; Clinical Center for HIV/AIDS, Beijing Ditan Hospital, Capital Medical University, Beijing, People's Republic of China; Dermatology & STD Department, Chifeng Infectious Disease Prevention and Control Hospital, Inner Mongolia, Chifeng, People's Republic of China; Center for Infectious Diseases, Beijing Youan Hospital, Capital Medical University, Beijing, People's Republic of China; Center for Infectious Diseases, Beijing Youan Hospital, Capital Medical University, Beijing, People's Republic of China; Center for Infectious Diseases, Beijing Youan Hospital, Capital Medical University, Beijing, People's Republic of China; Chinese Association of STD and AIDS Prevention and Control, Care and Treatment Committee, Beijing, People's Republic of China

**Keywords:** antiretroviral therapy, bictegravir, doravirine, noninferiority, real-world, switch therapy

## Abstract

**Background:**

We compared the effectiveness and safety profiles of doravirine, lamivudine, tenofovir disoproxil fumarate (DOR/3TC/TDF) with bictegravir, emtricitabine, tenofovir alafenamide fumarate (BIC/FTC/TAF) in people with HIV (PWH) who had achieved virological suppression on efavirenz (EFV)-based antiretroviral regimens.

**Methods:**

This study was a single-center, real-world, prospective observational cohort study. The main inclusion criteria: PWH aged ≥18 years who had received an EFV-containing regimen for ≥6 months and achieved confirmed virological suppression. Participants were stratified according to clinical decisions to switch to DOR/3TC/TDF or BIC/FTC/TAF. The primary effectiveness end point was the proportion of participants with HIV-1 RNA ≥50 copies/mL at week 48, with a preset 4% noninferiority margin.

**Results:**

A total of 349 participants received at least 1 dose of study drugs (142 in DOR group, 207 in BIC group). At 48 weeks, 2 (1.4%) in the DOR group and 1 (0.5%) in the BIC group had HIV-1 RNA ≥50 copies/mL (estimated treatment difference [ETD], 1.0%; 95% CI, −1.6% to 3.7%), establishing noninferiority. In the BIC group, mean CD4 counts decreased significantly by ∼76.5 cells/µL at week 48 (95% CI, −111.801 to −41.218; *P* < .001) compared with baseline. Over 48 weeks, adverse event rates were comparable between the 2 groups (*P* = .758). At week 48, the BIC group exhibited a baseline-adjusted mean increase of 0.269 mmol/L in total cholesterol (TC) and 0.171 mmol/L in low-density lipoprotein cholesterol (LDL-C), while the DOR group demonstrated a mean reduction of 0.453 mmol/L in triglycerides (TG), 0.412 mmol/L in TC, and 0.241 mmol/L in LDL-C relative to baseline. All β values for the group–time interaction terms were negative (*P* < .001). The change in body weight from baseline to week 48 in the DOR group was 1.8 kg lower than that in the BIC group (95% CI, −2.474 to −1.114; *P* < .001).

**Conclusions:**

In previously virologically suppressed PWH on an EFV-based regimen, the switch to DOR/3TC/TDF maintained virological suppression noninferior to that of BIC/FTC/TAF, with favorable metabolic profiles.

Since the widespread use of antiretroviral therapy (ART), HIV-1 infection has shifted from a fatal disease to a manageable chronic one. Yet, as patients’ survival extends, long-term medication's cumulative toxicity, metabolic complications, and quality of life issues have grown more prominent [1–5]. This has driven clinical practice from “simple viral suppression” to “comprehensive health management,” making regimen optimization for treatment-experienced patients key—to sustain virologic suppression, reduce adverse reactions, and improve long-term prognosis [6]. Integrase strand transfer inhibitors (INSTIs), with potent antiviral activity and a high resistance barrier, are recommended by international guidelines as first-line or switch options [7]. INSTI-based regimens like bictegravir, emtricitabine, tenofovir alafenamide fumarate (BIC/FTC/TAF) have shown excellent virologic response in large trials [8]. However, recent studies link INSTIs to higher risks of significant weight gain, dyslipidemia, and insulin resistance [9, 10]—abnormalities that may offset its virologic benefits, especially in patients with obesity or metabolic syndrome. The US Department of Health and Human Services (DHHS) suggests that treatment-experienced patients with metabolic syndrome or high cardiovascular risk switch to regimens with fewer metabolic impacts [11].

Doravirine (DOR), a new-generation non-nucleoside reverse transcriptase inhibitor (NNRTI), reduces traditional NNRTI's resistance risk and central nervous system (CNS) toxicity via optimized molecular structure and may have metabolic neutrality [12–14]. To date, clinical evidence directly comparing INSTIs and NNRTIs in treatment-experienced patients remains limited, particularly lacking in-depth metabolic analysis in the Chinese population. This study compares the effectiveness and metabolic impacts of doravirine, lamivudine, tenofovir disoproxil fumarate (DOR/3TC/TDF) and BIC/FTC/TAF in virologically suppressed people with HIV (PWH), exploring optimized switch therapy strategies.

## METHODS

### Ethical Approval

This study was approved by the Ethics Committee of Beijing Youan Hospital, Capital Medical University (No. 2022-086), and was registered on the HMA-EMA Catalogues of real-world data sources and studies (Registration Number: EUPAS103993). Informed consent was obtained from all participants.

### Study Design and Participants

This real-world, prospective observational cohort study was conducted at the Infection Center Outpatient Department of Beijing Youan Hospital, Capital Medical University. From May 16, 2023, to January 31, 2024, PWH meeting inclusion criteria were stratified according to clinical scenarios. Jointly decided by clinicians and participants, they switched to either the DOR group (DOR/3TC/TDF) or the BIC group (BIC/FTC/TAF). Baseline demographic and clinical characteristics of the 2 groups are summarized in Supplementary Table 1.

Main inclusion criteria: PWH aged ≥18 years; received efavirenz (EFV)-containing NNRTI regimen for ≥6 months with confirmed virological suppression HIV-1 RNA <50 copies/mL; willing and eligible to switch to either DOR/3TC/TDF or BIC/FTC/TAF under routine clinical decision-making. Exclusion criteria: history of nucleoside reverse transcriptase inhibitor (NRTI), NNRTI, or INSTI resistance mutations or treatment failure; hepatitis C virus (HCV) coinfection requiring antiviral treatment, unstable treated opportunistic infections or tumors (eg, active pulmonary tuberculosis, Kaposi's sarcoma) at switch; pregnant/lactating women; allergy to study drug components.

### Data Collection and Follow-up

Demographic data (age, sex, payment methods) and clinical data (ART initiation time, prior ART regimen, switch reasons, coinfections, comorbidities) were collected from electronic medical records. Clinical follow-up occurred at baseline (switch) and 12, 24, 36, and 48 weeks. At each visit, weight was measured and lab tests were done (complete blood count, liver/renal function, random blood glucose, lipid profile, uric acid [UA], urine routine, urine-specific protein). Plasma HIV-1 RNA, CD4 counts, and CD4/CD8 ratio were assessed at baseline, 24 weeks, and 48 weeks. All evaluations followed the study protocol without extra tests. Lipid classification referred to the Chinese Guidelines for Lipid Management (2023) [15], based on low-risk population standards for atherosclerotic cardiovascular disease (ASCVD) primary prevention. Body mass index (BMI) was categorized as follows: underweight <18.5 kg/m², normal 18.5–24.9 kg/m², overweight 25.0–29.9 kg/m², obesity ≥30 kg/m² [16].

The study terminated if participants modified ART postrecruitment, were lost to follow-up, or died. For participants with plasma HIV-1 RNA ≥50 copies/mL, viral load was retested within 4 weeks. If retesting yielded a result ≥200 copies/mL, participants withdrew and underwent genotypic resistance testing; if retesting yielded a result of 50–199 copies/mL (excluding poor adherence), withdrawal was decided by the research team based on clinical assessment.

### Adherence Assessment

Adherence was assessed using a pill count–based quantitative method; at each follow-up visit, the adherence rate was calculated by first determining the total prescribed dose for the follow-up period (based on daily administration frequency, pills per dose, and follow-up duration), then counting the remaining pills (excluding nonconsumption factors such as loss or damage), and finally calculating the ratio of actual consumed dose (total prescribed dose minus remaining pills) to total prescribed dose. Participants with a pill count–based adherence rate ≥90% were defined as having good adherence.

### Outcomes

The primary effectiveness end point was the proportion of participants with plasma HIV-1 RNA ≥50 copies/mL at week 48 (per the US Food and Drug Administration snapshot algorithm [17]). The key secondary effectiveness end point was the proportion of participants with plasma HIV-1 RNA <50 copies/mL at week 48 (same algorithm) plus virological effectiveness in subgroups (gender, age, CD4 counts, prior NNRTI ART usage time). Immunological effectiveness end points included changes in CD4 counts and CD4/CD8 ratio compared with baseline at weeks 24 and 48. Safety outcomes were assessed in all participants as treated, including the incidence of adverse events, weight changes, and abnormal laboratory indicators such as liver and kidney dysfunctions and lipid disorders during treatment.

### Statistical Analysis

This study aimed to confirm if switching to DOR/3TC/TDF was noninferior to BIC/FTC/TAF in virologically suppressed PWH (previously on NNRTI-based regimens), as measured by the proportion with HIV-1 RNA ≥50 copies/mL at week 48. Effectiveness was analyzed through modified intention-to-treat (mITT) and per-protocol (PP) analyses. Assuming 3% of participants in both groups would have plasma HIV-1 RNA ≥50 copies/mL at week 48, 300 non–randomly assigned participants (∼150 per group) were included to achieve ≥80% power (β = 0.2) for detecting noninferiority (4% margin for the primary end point), with a 1-sided α of 0.025% and a 10% dropout rate. For the primary end point, the between-group estimated treatment difference (ETD) was analyzed using the Cochran-Mantel-Haenszel (CMH) method. Noninferiority was established if the upper limit of the 95% CI was <4%. A similar analysis was conducted for key secondary end point (HIV-1 RNA <50 copies/mL), with noninferiority confirmed if the lower limit of the 95% CI was >–7%. Safety outcomes were evaluated in all treated participants. Abnormally distributed continuous variables used the Mann-Whitney *U* test. Categorical variables used the Pearson chi-square or Fisher exact test. Differences in indicators (eg, CD4 counts) between groups and across follow-up time points were analyzed using generalized estimation equations (GEEs). Statistical analyses were analyzed with SPSS 26.0 (IBM Corp., Armonk, NY, USA) and Python 3.9.0 (Python Software Foundation, https://www.python.org), with 2-tailed *P* values <.05 considered significant.

## RESULTS

From May 16, 2023, to January 31, 2024, 385 participants were screened. Thirty-six failed screening due to exclusion criteria (n = 2), not meeting inclusion criteria (n = 21), refusal (n = 12), and 1 in the BIC group had no drug exposure. A total of 349 participants received at least 1 dose of study drugs, 142 switched to DOR/3TC/TDF, and 207 switched to BIC/FTC/TAF. In the DOR group, 131 (92.3%) completed 48-week follow-up. Five switched regimens due to adverse reactions (n = 2) or economic reasons (n = 3), 1 discontinued, 1 was lost to follow-up, and 4 had no virological data. In the BIC group, 191 (92.3%) completed follow-up. Eight switched regimens due to adverse reactions (n = 3) or economic reasons (n = 5), 1 was lost to follow-up, and 5 had no virological data (Supplementary Figure 1). Both groups achieved good adherence, with pill count–based rates ≥90%. Most were male (344, 98.6%) with a median age (interquartile range [IQR]) of 36.0 (32.0–43.0) years. Forty-six (13.2%) were ≥50 years old. The median ART duration before enrollment (IQR) was 7.4 (5.4–8.6) years. Except for the switch reasons (renal-related indicators), the baseline characteristics were generally balanced between the 2 groups (Supplementary Table 1).

**Figure 1.**
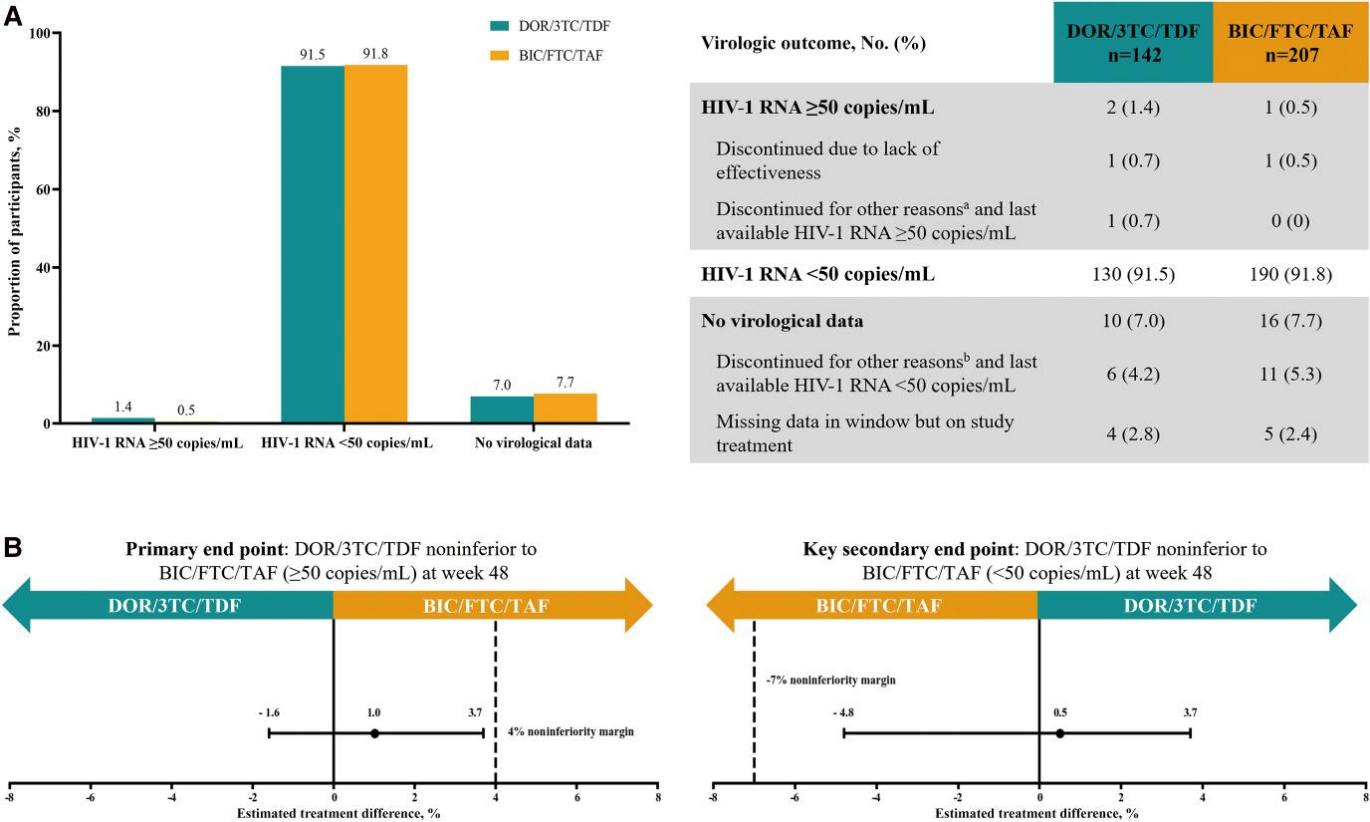
*A*, Virological outcomes at week 48 in the modified intention-to-treat population. *B*, Estimated treatment differences in the primary end point and key secondary end points. Data are presented as No. (%). The 95% CI for estimated treatment difference was calculated using the Cochran-Mantel-Haenszel method adjusted for non-nucleoside reverse transcriptase inhibitor usage time (<5 vs ≥5 and <8 vs ≥8 years). ^a^Due to poor compliance and self-discontinuation of medication. ^b^Other reasons include loss to follow-up, adverse events, or modified ART. Abbreviations: BIC/FTC/TAF, bictegravir/emtricitabine/tenofovir alafenamide fumarate; DOR/3TC/TDF, doravirine/lamivudine/tenofovir disoproxil fumarate.

At week 48, the noninferiority of switch to DOR/3TC/TDF was established compared with switch to BIC/FTC/TAF. HIV-1 RNA ≥50 copies/mL was 1.4% (DOR) vs 0.5% (BIC) (Figure 1*A*), the adjusted ETD was 1.0% (95% CI, −1.6% to 3.7%; upper CI <4% margin) (Figure 1*B*). The PP analysis was similar (adjusted ETD, 1.0; 95% CI, −1.8 to 3.9). Throughout the entire 48-week treatment process, both groups maintained high virological suppression. HIV-1 RNA <50 copies/mL was 91.5% (130/142) in the DOR group and 91.8% (190/207) in the BIC group (0.5%; 95% CI, −4.8% to 3.7%; noninferiority) (Figure 1*A, B*). Subgroup analysis showed no effectiveness differences regardless of baseline factors (Supplementary Figure 2*A, B*). GEE analysis showed that the time effect had similar effects on CD4 counts between the 2 groups (*P* = .079). In the BIC group, compared with baseline, the mean CD4 count at week 24 decreased significantly by ∼53.6 cells/µL (95% CI, −91.393 to −15.756; *P* = .005). At week 48, the mean CD4 count exhibited a significant reduction of roughly 76.5 cells/µL (95% CI, −111.801 to −41.218; *P* < .001) (Table 1). The CD4/CD8 ratio showed similar results (Table 1).

**Table 1.** Generalized Estimating Equations Analysis of Associations Between Treatment Group, Follow-up Time, and Changes in CD4 Count and CD4/CD8 Ratio

Variable	β	95% CI		Wald *χ*^2^	*P* Value
CD4 counts	…	…	…		
Time = 4 vs 0	−76.510	−111.801	−41.218	18.055	.000
Time = 2 vs 0	−53.575	−91.393	−15.756	7.709	.005
Group = DOR vs BIC	5.107	−59.028	69.243	0.024	.876
Time = 4 vs 0 * group = DOR vs BIC	49.410	−5.680	104.500	3.090	.079
Time = 2 vs 0 * group = DOR vs BIC	30.587	−35.660	96.834	0.819	.365
CD4/CD8 ratio	…	…	…		
Time = 4 vs 0	−0.206	−0.348	−0.064	8.030	.005
Time = 2 vs 0	−0.283	−0.422	−0.145	16.001	.000
Group = DOR vs BIC	−0.172	−0.341	−0.002	3.952	.047
Time = 4 vs 0 * group = DOR vs BIC	0.116	−0.040	0.271	2.117	.146
Time = 2 vs 0 * group = DOR vs BIC	0.091	−0.069	0.251	1.236	.266

Time 0 = baseline; Time 2 = week 24; Time 4 = week 48.

Abbreviations: BIC, bictegravir; DOR, doravirine.

By week 48, no participants in the BIC group met genotypic resistance testing criteria. Of 2 participants in the DOR group, 1 discontinued due to poor adherence, tested susceptible to NNRTIs, NRTIs, INSTIs, and re-achieved virological suppression after restarting treatment. The other had confirmed HIV-1 RNA ≥200 copies/mL but did not return for testing. All 13 participants who switched ART and withdrew had HIV-1 RNA <50 copies/mL.

During the 48-week follow-up, the adverse event rates were comparable between the 2 groups, with no statistically significant difference observed (*χ*^2^ = 0.149; *P* = .758). In the DOR group, 15.5% reported adverse reactions, including headache (n = 3), insomnia (n = 5), anxiety (n = 4), fatigue (n = 4), nausea/dysphagia (n = 4), and abdominal distension/diarrhea (n = 2). In the BIC group, 14.0% reported adverse reactions, including headache (n = 5), insomnia (n = 6), anxiety (n = 5), fatigue (n = 3), nausea (n = 2), abdominal distension/diarrhea (n = 5), and weight gain (n = 3). Two participants in the DOR group and 3 in the BIC group withdrew from the study due to adverse events, but no patients discontinued ART specifically due to adverse events.

**Figure 2.**
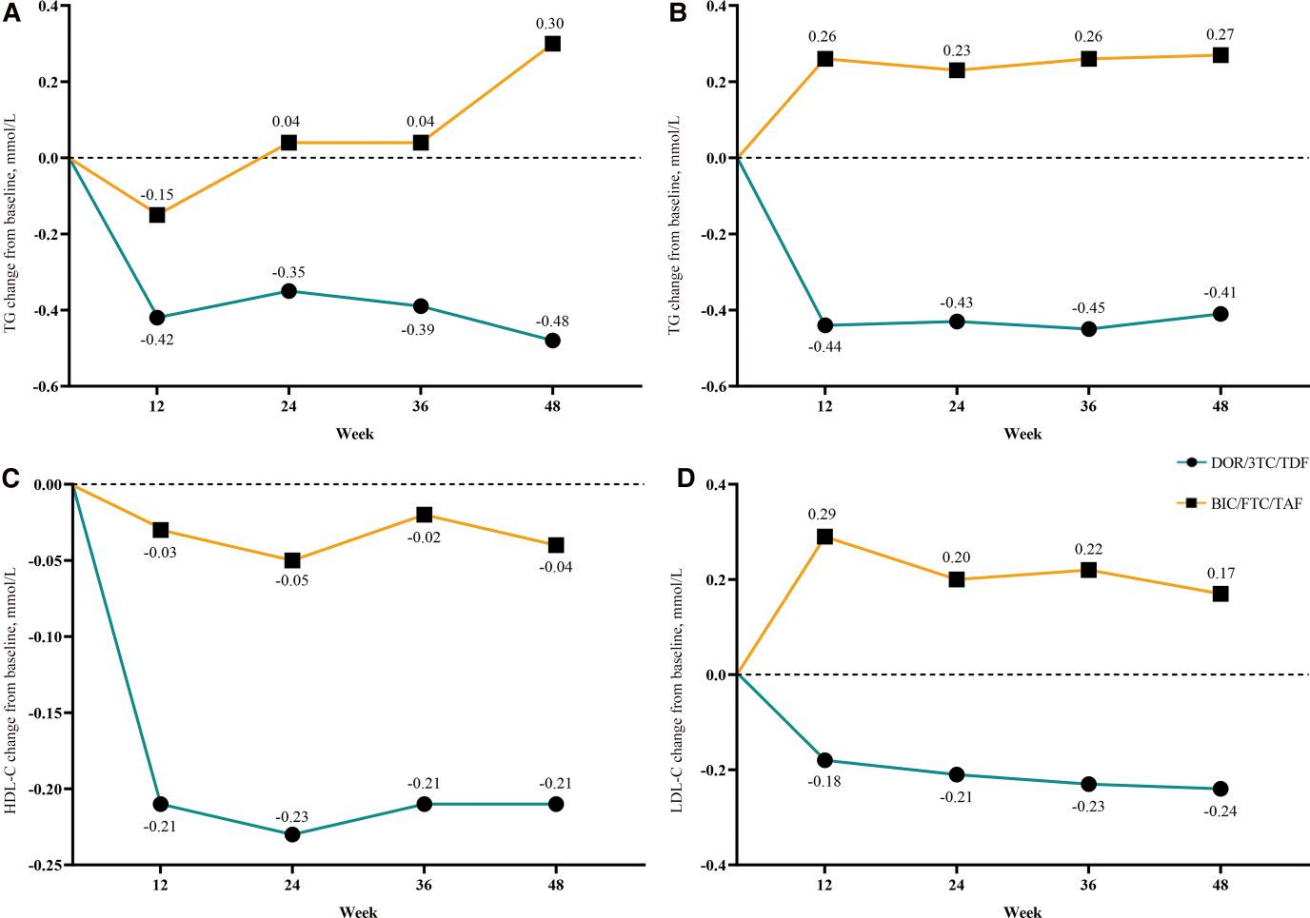
Changes from baseline in lipid parameters of the DOR and BIC groups over 48 weeks: (A) TG; (B) TC; (C) HDL-C; (D) LDL-C. Abbreviations: BIC, bictegravir; DOR, doravirine; HDL-C, high-density lipoprotein cholesterol; LDL-C, low-density lipoprotein cholesterol; TC, total cholesterol; TG, triglycerides.

During the study, only a very small number of patients in both groups received lipid-lowering medication (5 in the DOR group and 11 in the BIC group). At the end of follow-up, there was no statistically significant difference in the proportion of patients receiving lipid-lowering drugs between the 2 groups (*χ*^2^ = 0.035; *P* = .894). Figures 2*A–D*, presented as trend line plots, intuitively reflect the findings derived from GEE analyses, namely the 48-week changes in lipid profiles relative to baseline between the 2 groups. Compared with baseline, at 48 weeks, participants in the BIC group showed an increase in total cholesterol (TC) of ∼0.269 mmol/L (95% CI, 0.157 to 0.380; *P* < .001) and low-density lipoprotein cholesterol (LDL-C) of ∼0.171 mmol/L (95% CI, 0.072 to 0.271; *P* = .001). Significant group–time interaction (all β values were negative; *P* < .001) indicated different regimen effects on lipids. At week 48, the DOR group exhibited a mean reduction of ∼0.453 mmol/L in triglycerides (TG), 0.412 mmol/L in TC, and 0.241 mmol/L in LDL-C relative to baseline (Supplementary Table 2).

In the DOR group, the rates of dyslipidemia (assessed by TG ≥1.7 mmol/L, TC ≥5.2 mmol/L, and LDL-C ≥ 3.4 mmol/L) decreased from baseline values of 44.4%, 24.6%, and 26.1% to 27.9% (*χ*^2^ = 7.898; *P* = .005), 13.2% (*χ*^2^ = 5.735; *P* = .017), and 11.6% (*χ*^2^ = 9.075: *P* = .003) at week 48, respectively. In the BIC group, the rates of dyslipidemia (assessed by the same lipid parameters) changed from baseline values of 38.6%, 13.5%, and 12.1% to 39.5% (*χ*^2^ = 0.038; *P* = .844), 25.4% (*χ*^2^ = 8.772; *P* = .003), and 22.0% (*χ*^2^ = 6.810; *P* = .009) at week 48, respectively.

**Figure 3.**
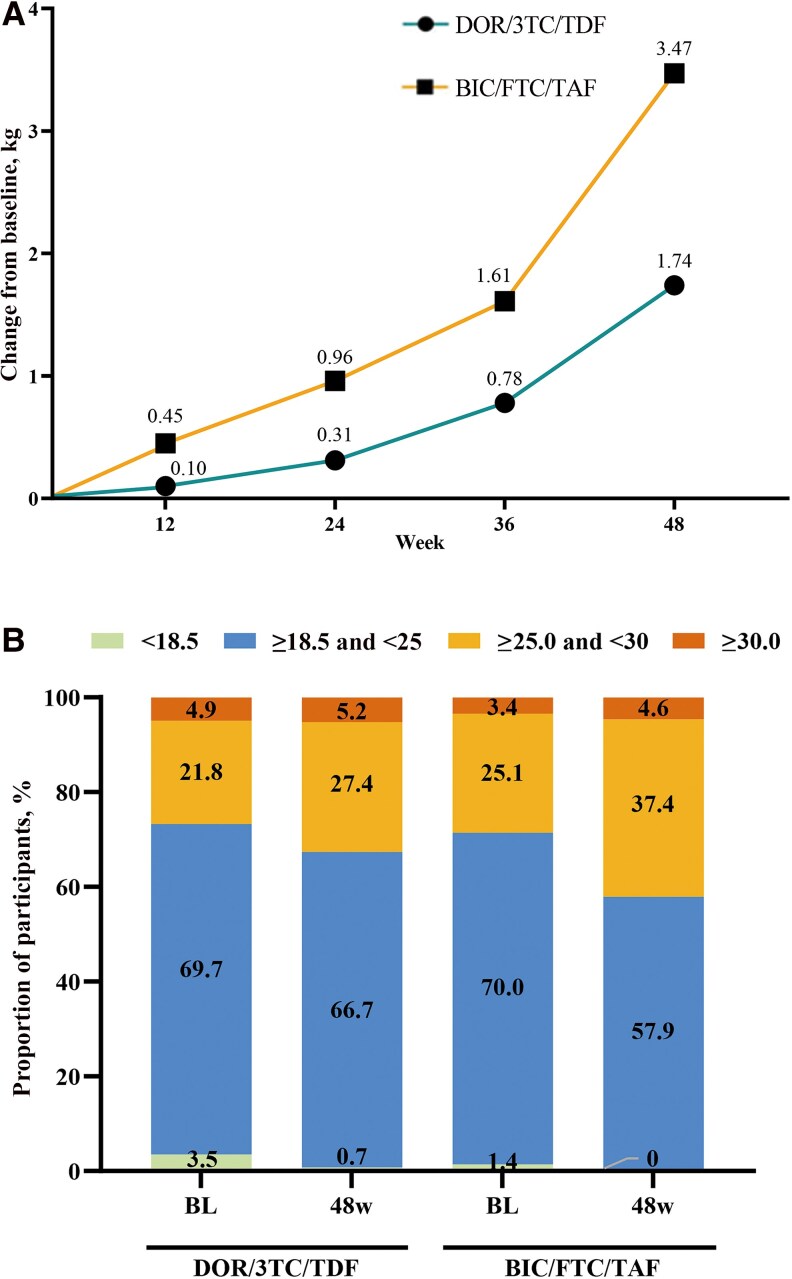
*A*, Changes from baseline in body weight of the DOR and BIC groups over 48 weeks. *B*, Evolution of BMI across 4 categories from baseline to week 48 in both groups. Abbreviations: BIC/FTC/TAF, bictegravir/emtricitabine/tenofovir alafenamide fumarate; BL, baseline; BMI, body mass index; DOR/3TC/TDF, doravirine/lamivudine/tenofovir disoproxil fumarate.

Additionally, Figure 3*A* shows the changes in body weight relative to baseline in both groups over the 48-week period. Follow-up time significantly affected weight (Wald *χ*^2^ = 320.324; *P* < .001). Significant group–time interaction (Wald *χ*^2^ = 28.183; *P* < .001) indicated different regimen effects on weight gain and inconsistent trends. At 48 weeks, participants in the BIC group had an average weight increase of 3.5 kg compared with baseline (95% CI, 3.080 to 3.964; *P* < .001). The change in body weight from baseline to week 48 in the DOR group was 1.8 kg lower than that in the BIC group (95% CI, −2.474 to −1.114; *P* < .001; GEE data not shown). Proportion changes of underweight, normal, overweight, and obesity are shown in Figure 3*B*.

During 48-week follow-up, changes in liver function were similar between the 2 groups (Supplementary Figure 3). For renal function, in the BIC group, compared with baseline, the mean Scr levels increased by approximately 7.6 µmol/L, 8.9 µmol/L, 10.6 µmol/L, and 11.6 µmol/L at weeks 12, 24, 36, and 48, respectively (all *P* < .001), with most changes occurring in early stages. After adjustment for baseline values, the increase in Scr over time in the DOR group was significantly lower than that in the BIC group (β = −3.957, −5.704, −5.451, and −6.121 at weeks 12, 24, 36, and 48; all *P* < .001) (Supplementary Table 3). The change in estimated glomerular filtration rate (eGFR) was the opposite (Supplementary Table 3). Both groups had minimal changes in serum calcium, phosphorus, cystatin C, urine tests (protein, occult blood, glucose), and urinary-specific proteins (GEE data not shown).

## DISCUSSION

This single-center, nonrandomized observational study evaluated the virological effectiveness and safety of switching from EFV to either DOR/3TC/TDF or BIC/FTC/TAF in virologically suppressed PWH. The primary analysis confirmed the noninferiority of DOR/3TC/TDF relative to BIC/FTC/TAF at week 48, as evidenced by the upper limit of the 95% CI for the difference in HIV-1 RNA ≥50 copies/mL rates being well below the predefined 4% margin. Consistent with this primary finding, high virological suppression rates (HIV-1 RNA < 50 copies/mL) exceeding 90% were achieved in both groups over the 48-week follow-up. Adverse event rates were comparable between the 2 regimens, with no statistically significant between-group differences observed. Notably, the DOR/3TC/TDF regimen demonstrated favorable metabolic profiles compared with the BIC/FTC/TAF regimen. These findings are consistent with those of recent domestic studies [18], supporting NNRTIs’ metabolic benefits and providing evidence for individualized therapy in treatment-experienced patients.

Although NNRTIs’ resistance barrier is lower than INSTIs’, real-world studies have shown potent antiviral activity in treatment-experienced patients without NNRTI resistance mutations—no confirmed virological failure at 96 weeks after switch [14]. The DRIVE-SHIFT study found 144-week outcomes consistent with 48 weeks, confirming long-term virological suppression with DOR-based regimens [19]. Our study's data further supported DOR/3TC/TDF's reliability as a switch regimen.

BIC/FTC/TAF had advantages such as a high resistance barrier and potent inhibitory ability and is often recommended as the preferred for switch therapy [20]. The BICSTaR study showed that the virological suppression rate of BIC/FTC/TAF in treatment-experienced patients could reach 97% [21]. However, in patients without NNRTI or INSTI resistance history, both regimens were reasonable simplified options, and virological effectiveness differences were possibly offset by other clinical factors (such as tolerance and metabolic risk).

Notably, changes in CD4 counts after switch were quite intriguing. Both groups had a slight decrease, but CD4 count changes in the BIC group at 24 and 48 weeks after switch were statistically significant (*P* < .05). A retrospective study on DOR/3TC/TDF from Italy found, during 1-year follow-up after switch, that there was a statistically significant CD4 enhancement of ∼50 cells/mL, while maintaining virological suppression [22]. In the BICSTaR study, after 1 year of treatment with BIC/FTC/TAF, an increase in median CD4 counts of treatment-experienced participants by 13 cells/µL (*P* = .014) was observed [21]. Conversely, some real-world studies and clinical trials found no CD4 changes [13, 14, 18]. ART-related CD4 changes are affected by multiple factors, such as comorbidities. In our study, the proportion of comorbidities in the BIC group was slightly higher than that in the DOR group, but the difference was not statistically significant. Thus, the underlying mechanisms are hard to elucidate, despite our observed trend. In fact, treatment-experienced patients (especially those on ART for >5 years with virological suppression) exhibit minimal CD4 response to regimen switches. Moreover, all participants maintained sustained virological suppression throughout the 48-week follow-up period, and the magnitude of absolute CD4 count reduction (≤100 cells/μL) fell within the range of normal biological variation, which is not considered clinically meaningful according to current HIV clinical practice guidelines [11].

Additionally, our study compared metabolic outcomes of treatment-experienced patients switching to DOR/3TC/TDF or BIC/FTC/TAF, showing that new-generation NNRTIs may reduce metabolic risk while maintaining virological suppression. The BIC group exhibited significant lipid worsening over the study period (*P* < .001). In contrast, the DOR group showed marked reductions in both lipid parameter levels and dyslipidemia rates (*P* < .001). These differences might have potential implications for long-term clinical management. Prior studies have demonstrated that INSTIs can induce dyslipidemia by directly promoting adipocyte differentiation or interfering with adipocyte mitochondrial function [23]. Specifically, BIC/FTC/TAF initiation or switch has been associated with increased metabolic disorders [24]. In contrast, switching to a DOR-based regimen yielded favorable lipid profiles: switching from rilpivirine (RPV) to DOR had minimal lipid changes, while switching from other NNRTIs/INSTIs to DOR improved lipids in a manner seemingly unrelated to TDF's “statin effect” [25]. Real-world studies further corroborated that switching from other NNRTIs (such as RPV or EFV) to DOR resulted in significant lipid improvements [26, 27]. This indicates that the differences in lipid parameters between the 2 groups are attributable not only to the pharmacological distinctions between TDF and TAF [28] but maybe also to the differential effects of the 2 core drugs, DOR and BIC.

Patients in the BIC group also experienced greater weight gain over the study period (*P* < .001), a finding consistent with prior evidence that INSTI-based regimens are linked to weight gain. For instance, the ADVANCE trial reported that 48-week weight gain with dolutegravir (DTG) + FTC/TAF was greater than that with NNRTI regimens [9]. A study focusing on ART initiation demonstrated that weight gain ≥10% from week 0 to 48 elevated the risk of metabolic syndrome (hazard ratio, 2.24) [29]. Weight gain might also adversely impact patient experience: Overweight and obesity are associated with self-image anxiety and poor treatment adherence in PWH. In our study, 3 patients in the BIC group switched regimens, again due to substantial weight gain. In contrast, the DOR group had significantly less weight gain (*P* < .001). Our findings align with the OPERA cohort study by Mounzer et al. [30], which reported weight loss benefits of doravirine in real-world settings, supporting the potential metabolic neutrality of doravirine. However, conflicting evidence exists: One study found that switching from INSTIs to DOR-based regimens resulted in a 2.6% weight loss within 1 year, but this change was not significant [31], while another important study showed that sustained weight gain in Black women switching from EFV/DTG to DOR was not reversed [32].

Dyslipidemia is typically insidious, often failing to garner sufficient attention from both patients and clinicians. Despite the relatively young age of PWH, nearly two-thirds are affected by dyslipidemia—a prevalence that is notably higher than that in HIV-negative individuals [33]. As the survival of PWH continues to improve, ASCVD has become the leading cause of death, necessitating targeted medical care [34]. Our study's lipid outcome data demonstrated that switching to the DOR/3TC/TDF regimen yielded significant improvements in both lipid parameter levels and dyslipidemia rates, offering a feasible strategy for the early management of dyslipidemia. Meanwhile, compared with the BIC/FTC/TAF regimen, the DOR/3TC/TDF regimen was associated with less weight gain, further enhancing its clinical appeal for patients at risk of metabolic complications. Collectively, these results support the integration of comprehensive metabolic risk assessment (encompassing both lipid profiles and body weight changes) into stable treatment switch decisions for virologically suppressed PWH. For patients with overweight/obesity, preexisting dyslipidemia, or high cardiovascular risk, DOR/3TC/TDF may thus represent a more favorable option than BIC/FTC/TAF, which aligns with the recommendations outlined in the 2023 European AIDS Clinical Society (EACS) guidelines [35].

Over the 48-week follow-up, participants in the BIC group had an increase in Scr, and the increase in Scr over time in the DOR group was significantly lower than that in the BIC group (all *P* < .001). This finding may be confounded by the specific metabolic properties of INSTIs, which are known to induce pseudohypercreatinemia rather than genuine renal injury. Notably, our results were consistent with those of the phase III SPRING study evaluating enovirine fumarate, lamivudine, and tenofovir dipyridamole (ANV/3TC/TDF) [18], which also demonstrated favorable renal safety profiles. Nevertheless, the nephrotoxicity of TDF is well recognized and exhibits a time dependency, a clinical concern that merits close monitoring in clinical practice. Therefore, in clinical decision-making, the benefits and risks of these 2 regimens should be comprehensively weighed.

Although our study provided evidence for the metabolic advantages of NNRTIs, the following issues still deserve attention. First, this was a single-center, nonrandomized observational design, which may introduce channeling bias—evidenced by baseline imbalances such as a higher proportion of patients with renal impairment in the BIC group—and restrict the generalizability of the findings. Additionally, the use of mITT and PP analyses may lead to selection bias: Nonrandom exclusion of patients without treatment exposure or efficacy data in the mITT cohort could distort treatment effects, while the PP cohort's restriction to protocol-adherent patients reflects an idealized clinical scenario rather than real-world practice, potentially overestimating treatment effectiveness and safety and further limiting external validity. Second, the number of virological failure events was small in both study groups, which limited the statistical power of the noninferiority comparison and may have increased the risk of Type II error. Third, the study population was predominantly male (98.6%), limiting generalizability to female PWH. Fourth, the 48-week follow-up duration cannot fully capture long-term metabolic and safety outcomes. Finally, our study lacked bone metabolism markers or imaging evaluations, so bone loss after switch was unevaluated. In subsequent studies, the dynamic changes of bone mineral density could be detected, and bone turnover markers could be jointly evaluated to identify bone metabolic abnormalities early.

In conclusion, at week 48, for virologically suppressed PWH on EFV-based ART, switching to DOR/3TC/TDF was not inferior to BIC/FTC/TAF in virological effectiveness. Adverse event rates were comparable between the 2 regimens. The DOR/3TC/TDF regimen had favorable metabolic profiles compared with the BIC/FTC/TAF regimen. Our results provide important evidence for precision management of HIV chronic diseases.

## Supplementary Material

ofaf808_Supplementary_Data

## Supplementary Data

Supplementary materials are available at Open Forum Infectious Diseases online. Consisting of data provided by the authors to benefit the reader, the posted materials are not copyedited and are the sole responsibility of the authors, so questions or comments should be addressed to the corresponding author.
