# Supplementary material for: Switch to Fixed Dose of Doravirine, Lamivudine, Tenofovir Disoproxil Fumarate Versus Bictegravir, Emtricitabine, and Tenofovir Alafenamide Fumarate in Virologically Suppressed Adults on Efavirenz-Based Regimens: 48-Week Results of a Real-world, Prospective, Observational Cohort Study
Source: Open Forum Infect Dis. 2026 Jan 20;13(1):ofaf808. doi: 10.1093/ofid/ofaf808 (PMC12817991; doi:10.1093/ofid/ofaf808)
Supplement: ofaf808_Supplementary_Data [file ofaf808_supplementary_data.zip › 4. Supplementary_Materials_Final_Clean.docx]

**Supplementary Figure 1. Participant recruitment flowchart.**

DOR/3TC/TDF: Doravirine/lamivudine/tenofovir disoproxil fumarate; BIC/FTC/TAF: Bictegravir/emtricitabine/tenofovir alafenamide fumarate; mITT: Modified intention-to-treat; PP: Per-protocol; ART: Antiretroviral therapy.

**Supplementary Figure 2. Forest plot of prespecified subgroup analyses: HIV-1 RNA < 50 copies/mL at week 48 (A) mITT population; (B) PP population.**

DOR/3TC/TDF: Doravirine/lamivudine/tenofovir disoproxil fumarate; BIC/FTC/TAF: Bictegravir/emtricitabine/tenofovir alafenamide fumarate; BMI: Body mass index; NNRTIs: Non-nucleoside reverse transcriptase inhibitors; CI: Confidence interval; mITT: Modified intention-to-treat; PP: Per-protocol.

**Supplementary Figure 3. Median changes from baseline in liver biomarkers at week 48.**

Data were median changes, with 95% CI. ALT: Alanine aminotransferase; AST: Aspartate aminotransferase; GGT: Gamma-glutamyl transferase; ALP: Alkaline phosphatase; TBIL: Total bilirubin; DBIL: Direct bilirubin; CI: Confidence interval.

**Supplementary Table 1. Baseline demographic and clinical characteristics.**

| Characteristics | DOR group  (*n* = 142) | BIC group  (*n* = 207) | Statistics | *P-*value |
| --- | --- | --- | --- | --- |
| Age, years, median (IQR) | 36.0 (32.0, 43.0) | 36.0 (32.0, 43.0) | -0.322 ^†^ | 0.747 |
| Age ≥ 50 years, n (%) | 19 (13.4) | 27 (13.0) | 0.008 ^*^ | 1.000 |
| Age < 50 years, n (%) | 123 (86.6) | 180 (87.0) |  |  |
| Sex |  |  | 1.980 ^**^ | 0.159 |
| Male, n (%) | 142 (100) | 202 (97.6) |  |  |
| Female, n (%) | 0 (0) | 5 (2.4) |  |  |
| Weight, kg, median (IQR) | 70.0 (65.0, 78.0) | 70.0 (65.0, 76.0) | 0.069 ^†^ | 0.945 |
| BMI, kg/m^2^, median (IQR) | 22.9 (21.5, 25.0) | 23.1 (21.5, 25.3) | 0.701 ^†^ | 0.483 |
| Any comorbidities, n (%) | ^a^ 12 (8.5) | ^b^ 30 (14.5) | 2.904 ^*^ | 0.096 |
| Serological positivity, n (%) |  |  |  |  |
| HBsAg | 5 (3.5) | 9 (4.3) | 0.149 ^*^ | 0.787 |
| Anti-HCV | 6 (4.2) | 10 (4.8) | 0.071 ^*^ | 0.804 |
| Anti-TP | 52 (36.6) | 80 (38.6) | 0.147 ^*^ | 0.737 |
| Previous ART usage time with NNRTIs, years, median (IQR) | 7.47 (5.05, 8.72) | 7.37 (5.50, 8.55) | -0.031 ^†^ | 0.975 |
| < 5, n (%) | 35 (24.6) | 40 (19.3) | 1.967 ^*^ | 0.386 |
| ≥ 5 and < 8, n (%) | 54 (38.0) | 92 (44.4) |  |  |
| ≥ 8, n (%) | 53 (37.3) | 75 (36.2) |  |  |
| Coadministered NRTIs, n (%) |  |  | - | 0.419 ^***^ |
| TDF+3TC | 124 (87.3) | 191 (92.3) |  |  |
| AZT/3TC | 16 (11.3) | 14 (6.8) |  |  |
| ABC+3TC | 1 (0.7) | 1 (0.5) |  |  |
| TAF+3TC | 1 (0.7) | 1 (0.5) |  |  |
| Previous use of lipid-lowering drugs, n(%) | 24 (16.9) | 33 (15.9) | 0,057 ^*^ | 0.883 |
| Reasons for switch, n (%) |  |  | 20.521 ^*^ | 0.001 |
| Simplify | 68 (47.9) | 88 (42.5) |  |  |
| Hyperlipidemia | 29 (20.4) | 31 (15.0) |  |  |
| CNS symptoms | 27 (19.0) | 33 (15.9) |  |  |
| Liver injury | 15 (10.6) | 23 (11.1) |  |  |
| Renal impairment | 0 (0) | 26 (12.6) |  |  |
| Others | 3 (2.1) | 6 (2.9) |  |  |
| Laboratory test |  |  |  |  |
| Blood |  |  |  |  |
| CD4 counts, cells/μL,  median (IQR) | 720 (552, 878) | 708 (524, 939) | -0.158 ^†^ | 0.875 |
| < 350 cells/μL, n (%) | 8 (5.6) | 15 (7.2) | 0.356 ^*^ | 0.663 |
| ≥ 350 cells/μL, n (%) | 134 (94.4) | 192 (92.8) |  |  |
| CD4/CD8 ratio, median (IQR) | 0.86 (0.58, 1.18) | 0.90 (0.65, 1.23) | 0.886 ^†^ | 0.376 |
| ALT, U/L, median (IQR) | 29.5 (21.6, 53.0) | 30.0 (22.0, 43.0) | 0.501 ^†^ | 0.617 |
| AST, U/L, median (IQR) | 26.5 (22.0, 36.0) | 28.0 (22.0, 34.0) | 0.319 ^†^ | 0.750 |
| TBIL, μmol/L, median (IQR) | 9.2 (7.6, 11.4) | 9.1 (7.3, 11.4) | 0.421 ^†^ | 0.674 |
| DBIL, μmol/L, median (IQR) | 3.4 (2.8, 4.3) | 3.5 (2.9, 4.3) | -0.588 ^†^ | 0.557 |
| GGT, U/L, median (IQR) | 40.5 (25.0, 77.3) | 36.0 (24.0, 65.0) | 1.312 ^†^ | 0.190 |
| ALP, U/L, median (IQR) | 94.0 (77.0, 107.0) | 94.0 (76.0, 114.0) | -0.462 ^†^ | 0.644 |
| CRE, μmol/L, median (IQR) | 68.0 (60.6, 74.0) | 68.0 (62.0, 78.0) | -2.075 ^†^ | 0.038 |
| eGFR, mL/min/1.73m^2^, median (IQR) | 117 (110, 123) | 113 (104, 122) | 2.155 ^†^ | 0.031 |
| Cys-C, mg/L, median (IQR) | 0.8 (0.8, 0.9) | 0.8 (0.7, 1.0) | -0.965 ^†^ | 0.334 |
| TG, mmol/L, median (IQR) | 1.6 (1.0, 2.5) | 1.4 (1.1, 2.2) | 0.763 ^†^ | 0.446 |
| TC, mmol/L, median (IQR) | 4.6 (3.9, 5.2) | 4.3 (3.8, 4.9) | 1.790 ^†^ | 0.075 |
| HDL-C, mmol/L, median (IQR) | 1.0 (0.9, 1.2) | 1.0 (0.9, 1.2) | 0.679 ^†^ | 0.497 |
| LDL-C, mmol/L, median (IQR) | 2.7 (2.2, 3.4) | 2.5 (2.2, 3.1) | 1.761 ^†^ | 0.078 |
| Urine |  |  |  |  |
| U-IgG, mg/L, median (IQR) | 5.0 (3.7, 8.7) | 5.6 (3.9, 10.0) | -1.785 ^†^ | 0.074 |
| U-TF, mg/L, median (IQR) | 2.3 (2.3, 2.3) | 2.3 (2.3, 2.3) | 0.083 ^†^ | 0.934 |
| U-mALB, mg/L, median (IQR) | 11.0 (10.9, 18.6) | 11.4 (11.0, 19.9) | -0.967 ^†^ | 0.333 |
| U-α1-MG, mg/L, median (IQR) | 15.7 (8.5, 29.4) | 21.8 (11.0, 35.8) | -2.434 ^†^ | 0.015 |
| U-β2-MG, mg/L, median (IQR) | 0.3 (0.2, 0.4) | 0.4 (0.2, 1.0) | -3.685 ^†^ | <0.001 |

### Data were shown as median (IQR) or *n* (%).

^*^ *χ*^2^ values; ^**^ continuity correction; ^***^ Fisher’s exact test; ^†^ *Z* values;

IQR: Interquartile range; DOR: Doravirine; BIC: Bictegravir; BMI: Body mass index; HBsAg: Hepatitis B surface antigen; HCV: Hepatitis C virus; TP: Treponema pallidum; ART: Antiretroviral therapy; NNRTIs: Non-nucleoside reverse transcriptase inhibitors; NRTIs: Nucleotide reverse transcriptase inhibitors; 3TC: Lamivudine; TDF: [Tenofovir disoproxil fumarate](http://muchong.com/html/201510/9467095.html); AZT: Zidovudine; ABC:Abacavir; CNS: Central nervous system; ALT: alanine aminotransferase; AST: aspartate aminotransferase; TBIL: total bilirubin; DBIL, direct bilirubin; GGT: gamma-glutamyl transferase; ALP: Alkaline Phosphatase; CRE: serum creatinine; eGFR: estimated glomerular filtration rate; Cys-C: cystatin C; TG: triglycerides; TC: total cholesterol; HDL-C: high-density lipoprotein cholesterol; LDL-C: low-density lipoprotein cholesterol; U-IgG: Urine immunoglobulin G; U-TF: Urine transferrin; U-mAlb: Urine microalbumin; U-α1-MG: Urine α1 - microglobulin; U-β2-MG: Urine β2 - microglobulin.

### ^a^ Included diabetes, hypertension, cerebral infarction, arrhythmia and mental system diseases (depression).

### ^b^ Included diabetes, hypertension, cerebral infarction, chronic kidney disease, arrhythmia, asthma, hyperthyroidism/hypothyroidism and mental system diseases (depression, obsessive-compulsive disorder).

**Supplementary Table 2. Generalized estimating equations analysis of associations between treatment group, follow-up time, and lipid profile changes.**

| Variable | | β | 95% CI | | | Wald *χ*^2^ | | *P*-value |
| --- | --- | --- | --- | --- | --- | --- | --- | --- |
| TG | |  |  | |  |  | |  |
| Time = 4 vs 0 | | 0.283 | -0.028 | | 0.593 | 3.178 | | 0.075 |
| Time = 3 vs 0 | | 0.003 | -0.201 | | 0.206 | 0.001 | | 0.979 |
| Time = 2 vs 0 | | 0.019 | -0.136 | | 0.175 | 0.059 | | 0.808 |
| Time = 1 vs 0 | | -0.101 | -0.257 | | 0.056 | 1.589 | | 0.208 |
| Group = DOR vs BIC | | 0.216 | -0.071 | | 0.503 | 2.181 | | 0.140 |
| Time = 4 vs 0 * Group = DOR vs BIC | | -0.736 | -1.118 | | -0.355 | 14.330 | | 0.000 |
| Time = 3 vs 0 * Group = DOR vs BIC | | -0.567 | -0.848 | | -0.286 | 15.639 | | 0.000 |
| Time = 2 vs 0 * Group = DOR vs BIC | | -0.448 | -0.705 | | -0.191 | 11.716 | | 0.001 |
| Time = 1 vs 0 * Group = DOR vs BIC | | -0.383 | -0.642 | | -0.124 | 8.393 | | 0.004 |
| TC | |  |  | |  |  | |  |
| Time = 4 vs 0 | 0.269 | | 0.157 | 0.380 | 22.282 | 0.000 | |  |
| Time = 3 vs 0 | 0.184 | | 0.058 | 0.309 | 8.243 | 0.004 | |  |
| Time = 2 vs 0 | 0.240 | | 0.132 | 0.347 | 18.961 | 0.000 | |  |
| Time = 1 vs 0 | 0.264 | | 0.161 | 0.367 | 25.272 | 0.000 | |  |
| Group = DOR vs BIC | 0.199 | | 0.013 | 0.385 | 4.379 | 0.036 | |  |
| Time = 4 vs 0 * Group = DOR vs BIC | -0.681 | | -0.840 | -0.521 | 69.963 | 0.000 | |  |
| Time = 3 vs 0 * Group = DOR vs BIC | -0.616 | | -0.785 | -0.447 | 50.971 | 0.000 | |  |
| Time = 2 vs 0 * Group = DOR vs BIC | -0.647 | | -0.799 | -0.496 | 69.957 | 0.000 | |  |
| Time = 1 vs 0 * Group = DOR vs BIC | -0.708 | | -0.858 | -0.557 | 85.352 | 0.000 | |  |
| HDL-C |  | |  |  |  |  | |  |
| Time = 4 vs 0 | -0.033 | | -0.068 | 0.002 | 3.409 | 0.065 | |  |
| Time = 3 vs 0 | -0.041 | | -0.078 | -0.005 | 4.900 | 0.027 | |  |
| Time = 2 vs 0 | -0.056 | | -0.085 | -0.026 | 13.593 | 0.000 | |  |
| Time = 1 vs 0 | -0.034 | | -0.063 | -0.006 | 5.542 | 0.019 | |  |
| Group = DOR vs BIC | 0.019 | | -0.040 | 0.078 | 0.391 | 0.532 | |  |
| Time = 4 vs 0 * Group = DOR vs BIC | -0.150 | | -0.199 | -0.100 | 35.107 | 0.000 | |  |
| Time = 3 vs 0 * Group = DOR vs BIC | -0.160 | | -0.209 | -0.110 | 39.429 | 0.000 | |  |
| Time = 2 vs 0 * Group = DOR vs BIC | -0.158 | | -0.206 | -0.109 | 41.146 | 0.000 | |  |
| Time = 1 vs 0 * Group = DOR vs BIC | -0.176 | | -0.219 | -0.134 | 65.624 | 0.000 | |  |
| LDL-C |  | |  |  |  |  | |  |
| Time = 4 vs 0 | 0.171 | | 0.072 | 0.271 | 11.347 | 0.001 | |  |
| Time = 3 vs 0 | 0.193 | | 0.084 | 0.302 | 12.126 | 0.000 | |  |
| Time = 2 vs 0 | 0.208 | | 0.114 | 0.303 | 18.554 | 0.000 | |  |
| Time = 1 vs 0 | 0.283 | | 0.191 | 0.374 | 36.684 | 0.000 | |  |
| Group = DOR vs BIC | 0.179 | | 0.008 | 0.351 | 4.217 | 0.040 | |  |
| Time = 4 vs 0 * Group = DOR vs BIC | -0.412 | | -0.553 | -0.272 | 33.137 | 0.000 | |  |
| Time = 3 vs 0 * Group = DOR vs BIC | -0.413 | | -0.562 | -0.263 | 29.358 | 0.000 | |  |
| Time = 2 vs 0 * Group = DOR vs BIC | -0.389 | | -0.518 | -0.260 | 34.858 | 0.000 | |  |
| Time = 1 vs 0 * Group = DOR vs BIC | -0.461 | | -0.590 | -0.331 | 48.604 | 0.000 | |  |

TG: Triglycerides; TC: Total cholesterol; HDL-C: High-density lipoprotein cholesterol; LDL-C: Low-density lipoprotein cholesterol; DOR: Doravirine; BIC: Bictegravir; CI: Confidence interval.

Time 0 = Baseline; Time 1 = Week 12; Time 2 = Week 24; Time 3 = Week 36; Time 4 = Week 48.

**Supplementary Table 3. Generalized estimating equations analysis for evaluating the effects of treatment groups and follow-up duration on renal biomarker changes.**

| Variable | β | 95% CI | | Wald *χ*^2^ | *P*-value |
| --- | --- | --- | --- | --- | --- |
| Scr |  |  |  |  |  |
| Time = 4 vs 0 | 11.576 | 10.298 | 12.853 | 315.450 | 0.000 |
| Time = 3 vs 0 | 10.592 | 9.334 | 11.850 | 272.415 | 0.000 |
| Time = 2 vs 0 | 8.858 | 7.578 | 10.139 | 183.926 | 0.000 |
| Time = 1 vs 0 | 7.610 | 6.370 | 8.850 | 144.639 | 0.000 |
| Group = DOR vs BIC | -3.187 | -5.629 | -0.744 | 6.537 | 0.011 |
| Time = 4 vs 0 * Group = DOR vs BIC | -6.121 | -8.008 | -4.234 | 40.420 | 0.000 |
| Time = 3 vs 0 * Group = DOR vs BIC | -5.451 | -7.298 | -3.603 | 33.437 | 0.000 |
| Time = 2 vs 0 * Group = DOR vs BIC | -5.704 | -7.546 | -3.861 | 36.814 | 0.000 |
| Time = 1 vs 0 * Group = DOR vs BIC | -3.957 | -5.786 | -2.128 | 17.975 | 0.000 |
| eGFR |  |  |  |  |  |
| Time = 4 vs 0 | -11.451 | -12.733 | -10.168 | 306.360 | 0.000 |
| Time = 3 vs 0 | -10.300 | -11.568 | -9.033 | 253.670 | 0.000 |
| Time = 2 vs 0 | -8.645 | -9.916 | -7.374 | 177.686 | 0.000 |
| Time = 1 vs 0 | -6.972 | -8.179 | -5.766 | 128.341 | 0.000 |
| Group = DOR vs BIC | 3.558 | 0.827 | 6.289 | 6.522 | 0.011 |
| Time = 4 vs 0 * Group = DOR vs BIC | 5.926 | 4.081 | 7.772 | 39.604 | 0.000 |
| Time = 3 vs 0 * Group = DOR vs BIC | 5.074 | 3.275 | 6.872 | 30.573 | 0.000 |
| Time = 2 vs 0 * Group = DOR vs BIC | 5.549 | 3.762 | 7.337 | 37.019 | 0.000 |
| Time = 1 vs 0 * Group = DOR vs BIC | 3.742 | 1.995 | 5.490 | 17.616 | 0.000 |

Scr: Serum creatinine; eGFR: Estimated glomerular filtration rate; DOR: Doravirine; BIC: Bictegravir; CI: Confidence interval.

Time 0 = Baseline; Time 1 = Week 12; Time 2 = Week 24; Time 3 = Week 36; Time 4 = Week 48.
